# Botulinum Neurotoxin-Producing Bacteria. Isn’t It Time that We Called a Species a Species?

**DOI:** 10.1128/mBio.01469-18

**Published:** 2018-09-25

**Authors:** Theresa Smith, Charles H. D. Williamson, Karen Hill, Jason Sahl, Paul Keim

**Affiliations:** a The Pathogen and Microbiome Institute, Northern Arizona University, Flagstaff, Arizona, USA; b BioScience Division, Los Alamos National Laboratory, Los Alamos, New Mexico, USA; VA Palo Alto Health Care System; Harvard Medical School

**Keywords:** *Clostridium botulinum*, botulism, botulinum neurotoxin, phylogenetic analysis, taxonomy

## Abstract

Botulinum neurotoxins (BoNTs) are produced by a diverse set of seven clostridial species, though alternate naming systems have developed over the last 100 years. Starting in the 1950s, a single-species taxonomy where any bacterium producing BoNT would be designated Clostridium botulinum was introduced. As the extreme diversity of these strains was recognized, a secondary system of taxonomic “groups” evolved. It became clear that these groups also had members that did not produce BoNT, and in some cases, they were given formal species names. Genomic analysis now clearly identifies species affiliations whether an isolate is toxigenic or not. It is clear that C. botulinum group nomenclature is no longer appropriate and that there are recognized species names for each clostridium. We advocate for the use of the scientific binomials and that the single-species group nomenclature be abandoned.

## Abstract (precis)

Botulinum neurotoxins (BoNTs) are produced by a diverse set of seven clostridial species, though alternate naming systems have developed over the last 100 years. Starting in the 1950s, a single-species taxonomy where any bacterium producing BoNT would be designated Clostridium botulinum was introduced.

## PERSPECTIVE

Botulinum neurotoxins are produced by at least seven bacterial groups that meet all the criteria of distinct species (1–5). The history of their discovery has been long and punctuated, which has resulted in convoluted and conflicting taxonomic nomenclature. However, careful examination of the taxonomic precedence reveals that a simple and rigorous naming system is already in place and merely needs to be universally adopted.

## THE HISTORY

Emile van Ermengem first described a botulinum neurotoxin (BoNT)-producing organism, which he named *Bacillus botulinus*. He likely believed that botulism was caused by a monospecific toxin produced by a single bacterial strain (6). This was proven erroneous on both counts in 1904 when G. Landmann described another bacterial toxin that had caused botulism following ingestion of contaminated bean salad (7). This was the first recorded case of botulism due to something other than preserved meat products, and it was determined that the toxin and the bacteria producing it differed from the van Ermengem strain (8). The two toxins were serologically distinct, and while van Ermengem’s organism was nonproteolytic, the Landmann strain was clearly proteolytic. In 1919, Georgina Burke separated the toxins from several U.S. BoNT-producing strains into two categories, which she designated toxin types A and B (9). The type A toxins appeared to be similar to that of the Landmann toxin, while the type B toxins were similar to the van Ermengem toxin. However, unlike with the van Ermengem strain, all of the U.S. isolates were proteolytic. Thus, it was shown that different bacterial strains may produce the same toxins and different toxins may be produced by the same bacterial strains, a truism that has since been reinforced numerous times.

At about this time, the Committee on Classification of the Society of American Bacteriologists proposed a genus name change separating the aerobic *Bacillus* species from the anaerobic *Clostridium* species (10), and this new genus designation came into popular usage in the 1920s (11–13).

As more toxin types were being discovered, additional differences among the strains were also noted. In 1922, type C toxin was identified from two different sources. Ida Bengtson isolated a toxin-producing bacterium from the larvae of *Lucilia caesar*, which had been implicated in chicken botulism (14). Similarly, H. R. Seddon reported a novel toxin type associated with cattle forage poisoning (15). While the toxin types were serologically similar, the bacterial strains exhibited different levels of proteolysis, prompting Seddon to designate his proteolytic isolates *Clostridium parabotulinum*, while the somewhat nonproteolytic strains of Bengtson remained Clostridium botulinum (15). The terms *C. parabotulinum* and C. botulinum continued to be used to differentiate proteolytic from nonproteolytic neurotoxin-producing clostridia for at least the next 30 years, after which A. R. Prevot and E. R. Brygoo (16) proposed designating any botulinum neurotoxin-producing organism Clostridium botulinum, based on that single overriding characteristic (J. Gunnison, personal correspondence; see also Fig. S1 in the supplemental material). This single-species designation based on toxin production has been problematic ever since.

10.1128/mBio.01469-18.1 FIG S1 Personal correspondence of Janet Gunnison concerning an international meeting at which the use of a single criterion for the nomenclature of BoNT-producing strains was adopted. (This letter was obtained from the ASM Archives at the University of Maryland, Baltimore County.) Download FIG S1, PDF file, 0.14 MB. Copyright © 2018 Smith et al. 2018 Smith et al. This content is distributed under the terms of the Creative Commons Attribution 4.0 International license.

## THE PROBLEM

Both neurotoxigenic and nonneurotoxigenic members that belong to each of the C. botulinum groups, as well as Clostridium argentinense, Clostridium baratii, and Clostridium butyricum, have been identified (2, 5, 17–20). The nontoxigenic isolates that were originally designated C. botulinum or *C. parabotulinum* do not fit the strict toxin species designation but nevertheless continued to be designated C. botulinum. In addition, there were repeated reports of clostridia that are distinctly different species but that nonetheless possessed the ability to produce botulinum neurotoxins (20–22). Within a few years of the 1953 nomenclature pronouncement, the bacterium that produced type G toxin was found to be sufficiently different from the others to be given its own species name, C. argentinense (20, 23). Additional discoveries of a BoNT/F-producing bacterial strain that was clearly the previously described C. baratii (21), followed by the isolation of a BoNT/E-producing *C. butyricum* strain (22), violated the convention that all BoNT-producing bacteria should be named C. botulinum.

While the single-species designation based upon neurotoxin production simplified BoNT-producing bacterial nomenclature, the strains producing these toxins exhibited a range of differences in their phenotypic characteristics, prompting some to adopt a “group” designation to distinguish among these bacteria for detection and identification purposes (Table 1) (1, 2, 24, 25). Group I included the proteolytic bacteria that had been named *C. parabotulinum*, while group II included nonproteolytic organisms that produced type B, E, and F toxins. Group III distinguished the type C- and D-producing organisms, and group IV was initially used to describe type G producers. Groups V and VI were briefly given as designators for BoNT-producing *C. baratii* and *C. butyricum* strains, respectively.

**TABLE 1:** Phenotypic characteristics of BoNT-producing clostridia

Traditional designation	Produces BoNT	Presence of:					Optimal growth temp (°C)	Proposed designation
		Lipase	Lecithinase	Gelatin liquefaction	Casein digestion	Glucose fermentation		
C. botulinum group I	Yes	**+**	**−**	**+**	**+**	**+**	35–40	C. parabotulinum
C. sporogenes	Yes	**+**	**−**	**+**	**+**	**+**		C. sporogenes
C. sporogenes	No	**+**	**−**	**+**	**+**	**+**		
C. botulinum group II	Yes	**+**	**−**	**+**	**−**	**+**	18–25	C. botulinum
C. botulinum group III	Yes	**+**	**−**	**+**	**−**	**+**	40	C. novyi sensu lato
C. novyi type A	No	**+**	**+**	**+**	**−**	**+**		
C. botulinum group IV	Yes	**−**	**−**	**+**	**+**	**−**	37	C. argentinense
C. subterminale	No	**−**	**−**	**+**	**+**	**−**		
C. hastiforme	No	**−**	**−**	**+**	**+**	**−**		
C. baratii	Yes	**−**	**+**	**−**	**−**	**+**	30–45	C. baratii
C. baratii	No	**−**	**+**	**−**	**−**	**+**		
C. butyricum	Yes	**−**	**−**	**−**	**−**	**+**	30–37	C. butyricum
C. butyricum	No	**−**	**−**	**−**	**−**	**+**		

## GENOME-BASED CLASSIFICATION

Although the single-species designation for C. botulinum has remained, there are now opportunities to improve and clarify the variation observed within and among these BoNT-producing bacteria. In particular, innovations in genome sequencing and analysis have revolutionized the way that bacteria can be classified. DNA-DNA hybridization (DDH) techniques were developed in the late 1960s (26), and DDH was accepted as a kind of “gold standard” for taxonomically characterizing bacteria. A review of DDH studies on BoNT-producing clostridia concluded that the genotypic and phenotypic groupings for these bacteria supported each other (2). C. botulinum group I members were closely related by DDH methodology, showing the >70% similarity that is considered to be the boundary for a determination to the species level. When tested, some nontoxigenic Clostridium sporogenes strains were discovered within this group, while other C*. sporogenes* strains were found to be unrelated to C. botulinum group I. Similarly, C. botulinum group II bacteria formed a distinct, closely related group. C. botulinum group III strains were directly linked to each other, and the pattern held with Clostridium novyi and Clostridium haemolyticum. C. botulinum group IV was designated a distinct species, namely, *C. argentinense*, which is related to some Clostridium subterminale strains. In addition, it was discovered that the toxigenic and nontoxigenic strains within both *C. baratii* and *C. butyricum* were otherwise indistinguishable.

**FIG 1:**
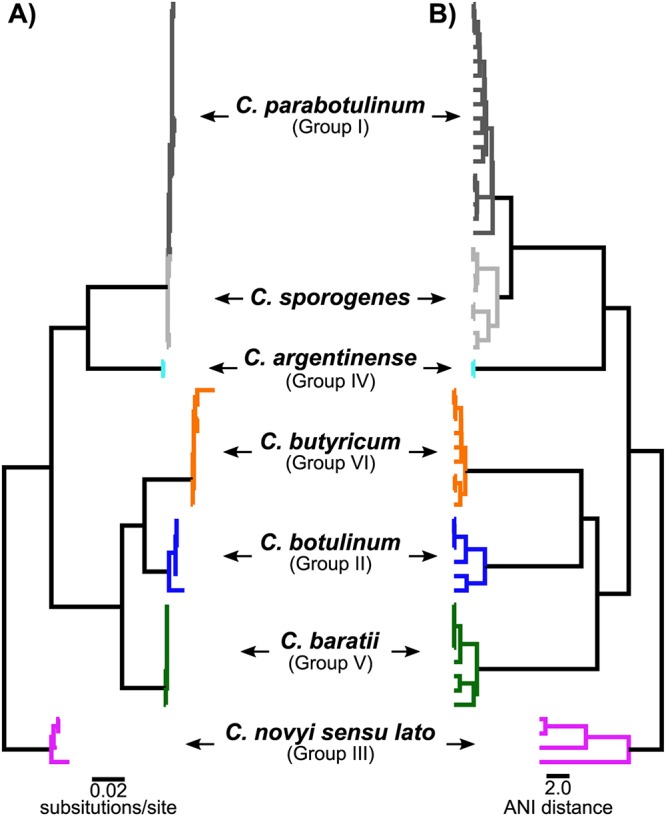
Dendrograms of BoNT-producing bacteria and closely related isolates. DNA sequence analysis of 54 isolates using 16S rRNA gene sequences (aligned with MUSCLE [32]) and maximum-likelihood analysis (IQ-TREE [33, 34]) (A) and using whole-genome sequences to estimate average nucleotide identity (ANI) with Mash (*k* = 21, *s* = 1,000,000) (30) followed by clustering with the unweighted pair group method with arithmetic mean (UPGMA) within QIIME (35) (B). Both methods separate the isolates into 7 taxonomic categories consistent with the historical species designations.

Due to technological difficulties surrounding DDH analysis, comparative analysis of 16S rRNA genes, highly conserved genes within all bacteria, succeeded this method (27, 28), and it was widely accepted as the next gold standard in this field. Importantly, analysis of 16S rRNA gene sequences in BoNT-producing clostridia confirmed the previous relationships based upon phenotypic characteristics and DDH techniques (Fig. 1A). (see also Table S1 in the supplemental material) While 16S rRNA gene analysis has proven extremely useful in bacterial evolutionary analysis, it is a single-gene analysis that is less than comprehensive and lacks discriminatory power at lower taxonomic levels.

10.1128/mBio.01469-18.2 TABLE S1 Clostridial strains analyzed in Fig. 1. Download Table S1, PDF file, 0.05 MB. Copyright © 2018 Smith et al. 2018 Smith et al. This content is distributed under the terms of the Creative Commons Attribution 4.0 International license.

Whole-genome sequencing (WGS) is now very common and provides the maximum level of genetic resolution for phylogenetic and systematic classification. WGS data analysis methods can target particular genome features and employ different evolutionary models to generate sophisticated insights into bacterial biology. One simple approach that merely uses genome similarity is pairwise average nucleotide identity (ANI) analysis (29). ANI analysis is an *in silico* phenetic methodology that compares bacterial genomic sequences for similarity, in much the same fashion that DDH did in the laboratory. While phenetic methods are considered weaker for phylogenetic inference, they work well for classification when the taxonomic groups are distinct and well separated in evolutionary time. This appears to be true for the BoNT-producing clostridia, and ANI analysis (estimations with Mash [30]) has confirmed earlier species designations and determined that earlier group designations are consistent with distinct species (Fig. 1B).

It is now well documented that there are seven distinct clostridial species capable of producing botulinum neurotoxins and that the botulinum toxin types produced are independent from the bacteria producing them. The idea that these bacterial groups are in fact several distinct species is now widely accepted and has historical precedence (1, 2, 5). The use of arbitrary group names that have no taxonomic status should cease and be replaced by Latin binomial nomenclature that has already been associated with these groups. We suggest the following: (i) that proteolytic C. botulinum group I species be referred to as *Clostridium parabotulinum*; (ii) that the Clostridium botulinum designation be restricted to the nonproteolytic group II organisms; (iii) that the BoNT/C- and BoNT/D-producing bacteria be included in the newly proposed species “*C. novyi sensu lato*” due to their documented close relationship with *C. novyi*, as Skarin et al. proposed (31), which would place these organisms genetically within the larger group of bacteria that includes classic *C. novyi* strains; and (iv) that the remaining BoNT-producing species (*C. argentinense*, *C. baratii*, *C. butyricum*, and *C. sporogenes*) retain their individual species names. As there are both toxic and nontoxic members within each of these species, the proposed changes provide the advantage that they do not rely solely on the expression of botulinum neurotoxin. For clarification, we further propose that BoNT-producing bacterial strains be additionally identified using the toxin type and/or subtype, such as “*C. parabotulinum* BoNT A1” or “*C. baratii* BoNT F,” to distinguish between toxic and nontoxic members. Adoption of these proposed species names will assist in clarification of the existing known organisms and provide a framework for the classification of future discoveries.
