# Supplementary material for: Botulinum Neurotoxin-Producing Bacteria. Isn’t It Time that We Called a Species a Species?
Source: mBio. 2018 Sep 25;9(5):e01469-18. doi: 10.1128/mBio.01469-18 (PMC6156192; doi:10.1128/mBio.01469-18)
Supplement: TABLE S1 [file mbo005184081st1.pdf]

SUPPLEMENTAL TABLE S1. Clostridial genomes analyzed in Figure 1.

Species	Toxin Subtype	Strain	Genome ID
C. parabotulinum (*C. botulinum* Group I)	HA+ A1	ATCC 19397	GCA_000017025.1
	HA+ A1	ATCC 3502	GCA_000063585.1
	HA+ A1	Hall	GCA_000017045.1
	HA-A1	CDC 297	GCA_000816945.1
	A2	Kyoto-F	GCA_000022765.1
	A2	Mauritius	GCA_002865765.1
	A2B5	CDC 1436	GCA_000817935.1
	A3	Loch Maree	GCA_000019545.1
	A5	H04402 065	GCA_000253195.1
	B1	okra	GCA_000019305.1
	B2	111	GCA_000829015.1
	B2	213B (ATCC 7949)	GCA_001273275.1
	B5a4	657	GCA_000020345.1
	F1	Langeland	GCA_000017065.1
	F4	Man216	GCA_002103875.1
	F5	SU0632	GCA_002103825.1
	NT	SU1575	GCA_001573935.1
C. botulinum (*C. botulinum* Group II)	B4	Eklund 17B	GCA_000020165.1
	E1	Beluga	GCA_000175335.1
	E3	Alaska E43	GCA_000020285.1
	E	NCTC 8266	GCA_000827935.1
	E	NCTC 8550	GCA_000827955.1
	F6	Eklund 202F	GCA_000789355.1
C. novyi sensu lato (*C. botulinum* Group III)	C	Stockholm	GCA_000219255.3
	D	1873	GCA_000175395.2
	CD	BKT015925	GCA_000204565.1
	NT	NT	GCA_000014125.1
C. argentinense (*C. botulinum* Group IV)	G	CDC 2741	GCA_000816675.1
	G	89G	GCA_002074155.1
C. baratii	F7	Sullivan	GCA_000789395.1
	F7	CDC 51267	GCA_001991075.2
	F7	771-14	GCA_000962755.1
	F7	694-15	GCA_002025785.1
	F7	796-15	GCA_002025805.1
	F7	693-15	GCA_002025815.1
	F7	695-15	GCA_002025875.1
	NT	2789STDY5834956	GCA_001405755.1
C. butyricum	NT	KNU-L09	GCA_001456065.2
	NT	JKY6D1	GCA_001465175.1
	NT	TOA	GCA_001646605.1
	NT	CDC 51208	GCA_001886875.1
	E4	5521	GCA_000171115.1
	E4	BL 5262	GCA_000182605.1
	NT	DKU-01	GCA_000355785.1
	NT	60E.3	GCA_000371625.1
	NT	DSM 10702	GCA_000409755.1
C. sporogenes	B2	Prevot 594	GCA_000816965.1
	B6	AM370	GCA_001573755.1
	B6	AM553	GCA_001573775.1
	B6	AM1195	GCA_002865785.1
	NT	ATCC 19404	GCA_001573855.1
	NT	ATCC 15579	GCA_000155085.1
	NT	DSM 795	GCA_001020205.1
	NT	NCIMB 10696	GCA_000973705.1
