# Supplementary material for: Botulinum Neurotoxin-Producing Bacteria. Isn’t It Time that We Called a Species a Species?
Source: mBio. 2018 Sep 25;9(5):e01469-18. doi: 10.1128/mBio.01469-18 (PMC6156192; doi:10.1128/mBio.01469-18)
Supplement: FIG S1 [file mbo005184081sf1.pdf]

1

UNIVERSITY OF CALIFORNIA
SAN FRANCISCO MEDICAL CENTER

SCHOOL OF MEDICINE
Department of Microbiology

November 20, 1963

Dr. L.S. McClung
Department of Bacteriology
Jordan Hall 438
Indiana University
Bloomington, Indiana 47405

Dear Dr. McClung:

Last year, I deposited all my representative strains of C. botulinum with the ATCC. They probably arrived there about the time of Dr. MacLennan's death and I have no idea whether they are in the collection. If not, I have portions of the same set of transfers. When you check the collection, please let me know if my strains are included and if they are pure and toxigenic.

It is good that you are working on the ATCC Clostridia. I have always been afraid that our type strains might get lost.

My feelings about the taxonomy of this group are divided. From the medical and public health point of view it is convenient and helpful to include all strains in one species. It is confusing to call the chief cause of human botulism in this country C. parabotulinum. In a weak moment, on a very hot afternoon in Rome, I gave in to the single species idea. Actually, I agree with you that the toxin should not override all other properties except for practical reasons. I still think that I can differentiate two species based on such simple criteria as microscopic appearance, odor, digestion of protein, and colony formation. I feel certain that the non-proteolytic strains would never be lumped with the proteolytic ones if it were not for the toxin. A non-toxigenic C. parabotulinum resembles C. sporogenes much more closely than it does C. botulinum, as you well know.

On the other hand, the committee at Rome felt that differences in ovolytic action were merely quantitative and some of the members had studied moderately proteolytic type C and D strains. Hence, they preferred to have only one species.

If you revive the old strains, I think they belong at the ATCC rather than with me provided that someone there knows how to maintain them. For one

thing, it is a lot of trouble to keep retesting them and, for another, I may retire before long.

Let me know what happens about the ATCC and your meeting with Dr. Sterne, please. Is he coming to San Francisco? When are you coming? It would be good to see you.

Sincerely,

Janet B. Gunnison

Janet B. Gunnison

JBG:ed
